# Expression and Cellular Immunogenicity of a Transgenic Antigen Driven by Endogenous Poxviral Early Promoters at Their Authentic Loci in MVA

**DOI:** 10.1371/journal.pone.0040167

**Published:** 2012-06-27

**Authors:** Toritse Orubu, Naif Khalaf Alharbi, Teresa Lambe, Sarah C. Gilbert, Matthew G. Cottingham

**Affiliations:** The Jenner Institute, University of Oxford, Oxford, United Kingdom; Ghent University, Belgium

## Abstract

CD8^+^ T cell responses to vaccinia virus are directed almost exclusively against early gene products. The attenuated strain modified vaccinia virus Ankara (MVA) is under evaluation in clinical trials of new vaccines designed to elicit cellular immune responses against pathogens including *Plasmodium* spp., *M. tuberculosis* and HIV-1. All of these recombinant MVAs (rMVA) utilize the well-established method of linking the gene of interest to a cloned poxviral promoter prior to insertion into the viral genome at a suitable locus by homologous recombination in infected cells. Using BAC recombineering, we show that potent early promoters that drive expression of non-functional or non-essential MVA open reading frames (ORFs) can be harnessed for immunogenic expression of recombinant antigen. Precise replacement of the MVA orthologs of *C11R, F11L, A44L* and *B8R* with a model antigen positioned to use the same translation initiation codon allowed early transgene expression similar to or slightly greater than that achieved by the commonly-used p7.5 or short synthetic promoters. The frequency of antigen-specific CD8^+^ T cells induced in mice by single shot or adenovirus-prime, rMVA-boost vaccination were similarly equal or marginally enhanced using endogenous promoters at their authentic genomic loci compared to the traditional constructs. The enhancement in immunogenicity observed using the *C11R* or *F11L* promoters compared with p7.5 was similar to that obtained with the mH5 promoter compared with p7.5. Furthermore, the growth rates of the viruses were unimpaired and the insertions were genetically stable. Insertion of a transgenic ORF in place of a viral ORF by BAC recombineering can thus provide not only a potent promoter, but also, concomitantly, a suitable insertion site, potentially facilitating development of MVA vaccines expressing multiple recombinant antigens.

## Introduction

The year 1983 saw the first descriptions of viral vectored vaccines, employing recombinant vaccinia virus to express foreign genes and elicit immune responses against various target pathogens [1]–[3]. Two different methods for expression of a cloned cDNA in vaccinia virus were employed in these inaugural studies, though both relied on insertion by homologous recombination in virus-infected cells. Whereas B. Moss’ group fused the cDNA of interest to a promoter prior to incorporation into vaccinia virus [2], [3], E. Paoletti and colleagues relied on endogenous transcriptional activity near the viral insertion locus [1], [4].

The disadvantage of using an endogenous promoter in the 1980s was that transgene expression by recombinants could be detected even when not adjacent to a transcriptional regulatory sequence [1], [5]. Using the technology and knowledge of the day, it was hard to avoid inserting extra sequence upstream of the transgene, so this phenomenon was attributed either to accidental presence of a sequence with weak promoter activity in the inserted sequence, or to formation of a fusion protein [5]. The linking of a cloned viral promoter and ORF of interest prior to insertion therefore became the standard method for generation of recombinant vaccinia virus [6]. Essentially the same technique, with variations, has been applied to other poxviruses [7]. Despite its sterling service in the eradication of smallpox, vaccinia virus lacks the improved human safety profile of attenuated derivatives such as NYVAC and MVA [8] or of avian poxviruses [9]. Yet as recombinants, these too have typically employed the p7.5 promoter, as in 1983, or one of a small number of other promoters with early/late activity, for example, H5 (previously called H6) [10], modified H5 (mH5) [11] and the short synthetic promoter (SSP) [12] to drive transgene expression. The cassette is generally still inserted into the traditional thymidine kinase (TK) locus, or into one of a similarly limited number of alternative loci: in MVA, into one of the sites of the large genomic deletions [13], or more recently into an intergenic region [14]–[15].

**Figure 1:**
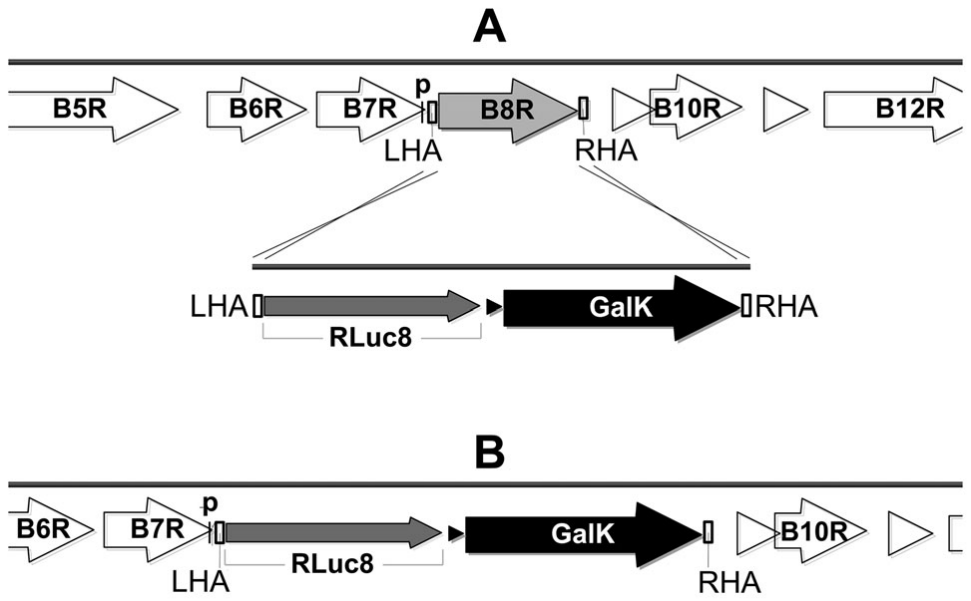
Schematic of transgene insertion at endogenous promoter driven locus of MVA, using *B8R* as an example. (A) Surrounding open reading frames (ORFs) in the MVA genome are indicated by white arrows, with *B8R* highlighted in grey. Black “p” above black bar indicates predicted *B8R* early promoter core region (see Table 2), overlapping with the *B7R* ORF. The left homology arm (LHA) and right homology arm (RHA) sequences (white boxes), each 50 bp in length, were added by PCR to the ends of a cassette comprising a model antigen, tPA-Pb9-rLuc8PV (narrow grey arrow) and the bacterial selectable marker *GalK* (black arrow), with its bacterial promoter (small black triangle). The LHA was designed to place the initiation codon of tPA-Pb9-rLuc8PV in the same position as that of the *B8R* ORF. Crossed lines indicate homology arm recombination events between targeting amplicon and MVA-BAC. (B) After recombineering of this 2.4 kb targeting amplicon into MVA-BAC to replace *B8R*, tPA-Pb9-rLuc8PV was placed under control of the *B8R* promoter.

The application of BAC recombineering technology to cloned poxviral genomes [16]–[18], coupled with recent transcriptomic studies [19]–[21], has allowed us to revisit the endogenous promoter method for expression of exogenous genes in a poxvirus. The increased precision of modern methodology allows direct replacement of a viral ORF with a coding sequence of interest, such that the initiation codon lies in exactly the same position relative to the upstream regulatory sequences (Figure 1). This simultaneously provides both a promoter and an insertion site for the transgene. Early transcription occurs within the virion core shortly after entry and cannot meaningfully be studied by transient co-transfection of reporter plasmids [22], so previous investigations of early promoter activity at the protein level used insertion of the promoter of interest at an unnatural locus, since there is no evidence that genomic context affects poxviral early transcription [23]. Here, we show that four early or “immediate-early” [20] (also referred to as E1.1 [19]) promoters, used at their authentic genomic loci in MVA, are able to drive early expression of a reporter gene equal to or exceeding the levels obtained using conventional recombinants with either the p7.5 or SSP promoters. By virtue of this approach, the insertion site is already provided with a promoter, and the promoter has its own insertion site, thus overcoming the need to find a suitable heterologous locus, and expanding the options available for transgene insertion, potentially of multiple recombinant antigens.

The protective efficacy of the first recombinant vaccinia virus vaccines was mediated by antibody responses (e.g. against hepatitis B and influenza [3], [24]) yet their ability to induce cytotoxic T lymphocyte responses was also recognized at the time [25]. Heterologous prime-boost vaccination regimens [26] improved CD8^+^ T cell induction and provided one of the most promising routes to development of desperately needed new vaccines against diseases that have resisted traditional vaccinological approaches, such as the major global killers HIV/AIDS, tuberculosis and malaria [27]. Recently, the combination of a recombinant chimpanzee adenovirus boosted by MVA expressing the same transgenic antigen has achieved unprecedented frequencies of vaccine-induced antigen-specific CD8^+^ T cells in humans [28]. Here, we show that expression of recombinant antigen from four endogenous early or “immediate-early” promoters in MVA elicits equal or slightly superior frequencies of specific CD8^+^ T cells compared to p7.5 or SSP in either single-shot or adenovirus-prime, MVA-boost vaccination regimens in mice. We also present an indirect comparison with mH5. Furthermore, insertion into these loci did not adversely affect viral growth and the recombinant viruses were genetically stable, indicating the applicability of the resurrected and improved endogenous-promoter method to vaccine design.

## Materials and Methods

### Model Antigen based on Renilla Luciferase

A cDNA encoding a variant of *Renilla reniformis* (sea pansy) luciferase, rLuc8, which exhibits improved stability and light output [29], was obtained from Dr Sanjiv Gambhir, Stanford University, USA. A poxviral early transcription termination motif (T5NT) was removed by PCR mutagenesis, such that the isoleucine at position 48 is encoded by ATC instead of ATT. We further modified the encoded protein by fusing two sequences to the N-terminus: the H2-K^d^ restricted murine CD8^+^ T cell epitope SYIPSAEKI (Pb9) from the *Plasmodium berghei* circumsporozoite protein [30] and the signal peptide comprising amino acids 1–28 of human tissue plasminogen activator (tPA). The sequence MDD linked tPA and Pb9 and the sequence GS linked Pb9 and rLuc8. A T5NT early termination sequence was placed immediately downstream of the tPA-Pb9-rLuc8PV open reading frame. The resulting construct, tPA-Pb9-rLuc8PV, encodes a secretable, Pb9-tagged version of rLuc, with enhanced extracellular stability [29], and suitable for poxviral early expression.

### Insertion into Endogenous Promoter Driven Loci of MVA-BAC

Construction and generation of MVA-BAC and generation of MVA deletion mutants using *GalK* recombineering [31] has been described previously [16]. To generate recombinant MVA (rMVA) viruses expressing tPA-Pb9-rLuc8PV under the control of viral promoters at their natural loci (Tables 1 and 2), we employed a modification of the *GalK* deletion method (Figure 1). A cassette was constructed using conventional PCR and restriction enzyme based cloning, comprising the tPA-Pb9-rLuc8PV open reading frame and the bacterial *GalK* resistance gene. This was amplified with Phusion (Finnzymes) as a targeting DNA for recombineering by using long oligonucleotide primers (Eurofins MWG Operon) to add 50 bp homology arms to the 5′ and 3′ ends. The primers were designed to delete the viral ORF wholly or partially (depending on the predicted effects of deletions on downstream genes) and to replace it with tPA-Pb9-rLuc8PV and the bacterial selectable marker. The homology arm immediately 5′ to the tPA-Pb9-rLuc8PV ORF was designed to place the initiator codon (ATG) of the inserted ORF at the same position as that of the deleted viral gene (Table 2). These targeting constructs were used for MVA-BAC recombineering as previously described [16]. *GalK* selection was used to facilitate removal of the marker and ‘recycling’ for insertion at a second locus, though we did not take advantage of this in the present paper.

**Table 1:** Loci selected for insertion of endogenous promoter driven transgene by replacement of MVA open reading frame (ORF).

MVA ORF	Vaccinia ortholog	Function in vaccinia virus	MVA-specific mutations	Promoter activity*	Position of ATG in GenBank U94848.1 [43]
*176R*	*B8R*	IFN-γ soluble receptor [63]	3′ inactivating truncation	Early (IE | E1.1)	157621 (top strand)
*041L*	*F11L*	Cell motility (RhoA inhibitor) [64]	Fragmented *(040L+041L)*	Early ( E | E1.1)	33771 (bottom strand)
*027L*	*K6L*	Unknown; fragmented putative monoglyceride lipase (*K6L+K5L*)	Fragmented; see Results	Early (IE | E1.1)	24694 (bottom strand)
*157L*	*A44L*	3β hydroxysteroid dehydrogenase [46]	1 amino acid substitution	Early (IE | E1.2)	15377 (bottom strand)
*005R*	*C11R*	Vaccinia viral growth factor (VGF) [65]	None	Early (IE | E1.1)	10203 (top strand)
*168R*	*B2R*	Unknown; fragmented (*B2R+B3R*)	Fragmented *(168R*+*169R)*	Early (IE | E1.2)	152144 (top strand)

* Designations in brackets refer to categorisation into immediate early (IE) or early (E) clusters by microarray analysis [20], and into the corresponding E1.1 and E1.2 clusters by deep sequencing [19], of vaccinia virus mRNA.

**Table 2:** Sequences of promoters, with transcriptional and translational (ATG) start sites shown capitalised, and the predicted early promoter core sequences underlined [19].

*B8R*	atattattcaaaatatgatttttaaaAatttaaaatatattatcacttcagtgacagtagtcaaataacaaacaacaccATG
*F11L* a	aaaaaagtgaaaaacaatattattttTatcgttggttgtttcactATG
*A44L*	gtaaaatagaataagtagtctgatattaTgagtggcagcaATG
*K6L* b	ataaaacataaaaataatatgatcatcaAacgaactgttaatattgatagttatataacgtgaatcATGagtgcaaactgtatgttcaatctggacaATG
*C11R*	atattactgaattaataatAtaaaattcccaatcttgtcataaacacacactgagaaacagcataaacacaaaatccatcaaaaATG
*B2R* b	cgataaaaattaaaaaaTaacttaatttattattgatctcgtgtgtacaaccgaaatcATGgcgatgttttacgcacacgctctcggtgggtacgacgagaatcttcATG
p7.5^c^	taaaagtagaaaatatattctaatttatTgcacggtaaggaagtagaatcataaagaacagt–MCS
SSP^c^	taaaaattgaaattttattttttttttttTgaatataaataa–MCS

a The italicised ‘t’ in *F11L* was mutated during recombineering to identity with vaccinia virus Western Reserve strain (VACV-WR).

b The second (downstream) ATG was used in the recombinant viruses described in the text. This is the ATG of the ORF as originally annotated in MVA [43]. The upstream ATG, however, likely represents the authentic translational start site in vaccinia virus, encoding a protein that is severely truncated in MVA by a small deletion (see text).

c Conventional insertion of promoter linked to ORF at the TK locus of MVA. Dashes indicate appendage of multiple cloning site (MCS).

### Insertion into TK Locus for Control Constructs using MVA-BAC

An rMVA expressing tPA-Pb9-rLuc8PV from the traditional promoter, p7.5, inserted at the thymidine kinase (TK) locus was constructed using pEP75TK and *AphAI* BAC recombineering as previously described [32]. We did not take advantage of this system’s *en passant* capability for *AphAI* removal in the present paper. The p7.5 promoter of pEP75TK was replaced with the short synthetic promoter, SSP [12], or the mH5 promoter [11] by standard PCR and restriction enzyme techniques. This construct was used in parallel to generate rMVA expressing tPA-Pb9-rLuc8PV under control of SSP.

### MVA-BAC Rescue and Propagation of rMVA

The recombineered MVA-BACs were rescued to recombinant MVA in BHK cells (obtained from ATCC via LGC Standards) using a fowlpox virus helper as previously described [16]. To avoid a second round of recombineering, and to establish viral viability at an early stage, the *GalK* or *AphAI* bacterial marker genes were not removed prior to rescue. BACs and derived viruses were checked for identity and purity by PCR and the sequences of the homology arms and transgenes were confirmed at both stages. BAC-derived rMVAs were plaque-picked three times to ensure purity, as a precautionary measure: carry-over of *GalK*-negative “hitch-hikers” is sometimes problematic in this positive metabolic selection system (this can alternatively, or additionally be addressed by repeated bacterial re-streaking on MacConkey indicator plates [33]). The viruses were amplified in 1500 cm^2^ of BHK cell monolayers, partially purified over sucrose cushions and titred in primary chicken embryo fibroblast (CEF) cells (obtained from the Institute for Animal Health, Compton Laboratory, UK) according to standard practice, and purity and identity were again verified by PCR. Since MVA-BAC has a GFP marker gene under control of the Fowlpox virus p4B promoter [16], all the rMVAs expressed GFP in addition to tPA-Pb9-rLuc8PV.

### Adenovirus Vectored Vaccine

An E1/E3-deleted chimpanzee adenovirus, ChAd63 [34], expressing TIP, a model epitope string antigen which also contains the Pb9 epitope [35], was constructed as previously described [36]. ChAd63-TIP was purified by CsCl gradient ultracentrifugation and titred by immunolabelling (ifu) using Cell Biolabs’ QuickTiter kit modified for 96-well plates.

### Luciferase Assays

For luciferase assays, a “spinoculation” protocol was used [37] in order to synchronize the infection and enable prior washing of the cells to remove rLuc activity in the inoculum (see Results). BHK cells (5×10^4^ cells/well) in flat-bottom 96-well microtitre plates were inoculated in duplicate with rMVAs at 1 pfu/cell. The plates were centrifuged at 650G for 1h at 0°C then washed three times with ice-cold DMEM containing 2% FCS, before being placed at 37°C in 150 µL per well of medium which optionally contained 40 µM cytosine arabinoside (AraC). A 20 µl aliquot of supernatant was taken immediately after washing, then at 1 h, 2 h, 4 h, 8 h and 24 h post-infection, at which time the cells were washed in PBS and lysed in a volume of 150 µL. The rLuc activity in 10 µL aliquots of these samples was quantified using the *Renilla* Luciferase Assay System (Promega) and a Varioskan Flash luminometer (Thermo).

### Mouse Immunogenicity

Female BALB/C mice aged 6 to 8 weeks were immunized intramuscularly (i.m.) in the tibialis muscles (total volume 50 µL) with a total of 10^6^ pfu of rMVA, or with 10^8^ ifu of ChAd63 followed eight weeks later with 10^6^ pfu of rMVA for the heterologous prime boost regimen. Mice were used in accordance with the UK Animals (Scientific Procedures) Act 1986 under project license number 30/2414 granted by the UK Home Office. For induction of short-term anaesthesia, animals were anaesthetised using vaporised IsoFlo^®^. Splenocytes were harvested seven (single-shot) or fourteen (prime-boost) days post-immunization for analysis by IFN-γ ELIspot or flow cytometry with intracellular cytokine staining (ICS), both as previously described [16], [36], using re-stimulation with 1 µg/mL Pb9 peptide [30]. In the absence of peptide restimulation, the frequency of IFN-γ^+^ CD8^+^ cells was <0.1% by flow cytometry or <50 sfc/10^6^ splenocytes by ELIspot.

### Viral Genetic Stability Assay

For serial passage, CEF cells in 25 cm^2^ flasks were inoculated with 100 µl of crude lysate (or initially with 1 pfu/cell) incubated until all cells were infected as determined by epifluorescence microscopy for GFP (2–3 days), and subjected to triple freeze thaw. The process was repeated 10 times, after which the viruses were titred on CEF cells. Note that all the BAC-derived rMVAs expressed GFP in addition to tPA-Pb9-rLuc8PV (see above). Titres fell within the range of 2.4 to 3.8 × 10^5^ pfu/mL. To determine what proportion of viruses retained expression of the model antigen tPA-Pb9-rLuc8PV, CEF cells in 150 cm^2^ flasks were infected at 0.001 pfu/cell and 2 days later, single GFP^+^ trypsinized cells were sorted into individual wells of 96-well plates using the CyCLONE robotic module of a MoFlo (Dako Cytomation) flow cytometer. These plates were seeded with 5 × 10^4^ BHK cells per well and 3 days later the wells were scored positive or negative for GFP by epifluorescence microscopy before quantification of rLuc activity in the cell lysates as above. Not all BHK wells were GFP^+^, owing to unavoidable errors in the MoFlo droplet identification combined with the imperfect correlation between presence of infectious virions and GFP positivity in the sorted CEF cells. Wells were scored positive or negative for rLuc based on a cut-off of three standard deviations above the geometric mean of the light units detected in GFP^–^ (i.e., uninfected) wells. Any genetic instability at the tPA-Pb9-rLuc8PV insertion locus would result in absence of luciferase activity in a GFP^+^ well.

### Viral Growth Rate Assay

The growth rates of rMVAs were determined by GFP fluorescence as previously described [32]. Briefly, BHK cells (seeded at 5×10^4^ cells/well) in a black-walled, clear-bottom 96-well plate were infected in duplicate with rMVAs at various multiplicities of infection and GFP fluorescence was quantified every 6 min for 36 h using a BMG FluoSTAR fluorimeter equipped with 37°C+5% CO_2_ incubation.

## Results

### Selection of Non-essential, Highly Expressed Genes for Replacement with a Transgenic ORF

To investigate promoter activity at the natural locus by deleting a non-essential MVA gene and replacing it with the ORF of interest, leaving the latter under control of the deleted gene’s natural promoter (Figure 1), we selected, amongst many possibilities, six non-essential loci in MVA (Table 1), on the basis of the following criteria.

First, we identified highly expressed early or “immediate-early” (IE) genes from the vaccinia virus microarray data of Assarsson *et al*. [20]. (At the time, the more recent deep RNA sequencing paper, in which IE genes were designated E1.1 [19], had not been published). Recombinant MVA and other poxviruses are attracting attention as vaccine vectors owing to their ability to induce antigen-specific CD8^+^ T cells [38]. Such responses to vaccinia virus infection are directed almost exclusively against early viral antigens [39]–[42]. We hypothesized that the early or IE promoters driving expression of these antigens would therefore be capable of eliciting high frequencies of CD8^+^ T cells against a recombinant antigen in MVA.

Second, since the viral ORF is deleted concomitant with insertion, it is important that the targeted gene is non-essential. From this point of view, MVA has the advantage that many of its genes were inactivated during attenuation by serial passage [43], [44]. Of the six selected genes (Table 1), three are fragmented, and *B8R* has a truncation that is known to inactivate the encoded IFN-γ binding protein [45]. The immunodominant CD8^+^ T cell epitope in C57BL/6 mice is encoded by *B8R* [40], making this gene a very strong candidate. Both *A44L* [46], [47] and *C11R* [48], [49] are virulence factors that do not affect vaccinia virus replication *in vitro;* and we have shown that deletion of *A44L* [16] or *C11R* (Cottingham *et al.*, unpublished data) does not affect MVA immunogenicity.

Third, we avoided ORFs with upstream regions carrying MVA-specific mutations, since these might affect promoter function compared to vaccinia virus (e.g. *F7L, K1L*). We did not exclude *F11L* on this basis, but instead reverted an MVA-specific substitution just upstream of the initiation codon back to identity with vaccinia virus (Table 2).

### Generation of Recombinant Viruses by MVA-BAC Recombineering

Recombineering primers were designed to insert a model reporter transgene, tPA-Pb9-rLuc8PV, into the selected loci (Table 1), replacing the viral ORF (Figure 1). This was done such that the ATG of the deleted MVA ORF was replaced with that of the transgene (Table 2), followed by the bacterial selectable marker *GalK*. Recombineering reactions were efficient in all cases, as was found previously [16]. As controls, we constructed traditional-style recombinants employing the p7.5 and SSP promoters to drive the same transgene at the TK locus, also using BAC recombineering [32]. These promoters were linked to the ORF by conventional ligation, so contained an intervening sequence containing part of the multiple cloning site. The BACs were rescued, amplified, and semi-purified, and the eight resulting viral preparations (Table 1) had yields ranging from 1.8 × 10^9^ to 5.4 × 10^9^ pfu/mL (final volume ∼0.5 mL from 1500 cm^2^ flask area), which lies in the expected range for conventional and BAC-derived MVA [32]. The purity and identity of the recombinant viruses were verified by PCR analysis (not shown). Insertion into each of the six candidate loci did not therefore affect the efficiency of BAC rescue or the viral productivity.

### MVA Transgene Expression in vitro Driven by Endogenous Promoters at Their Natural Loci

**Figure 2:**
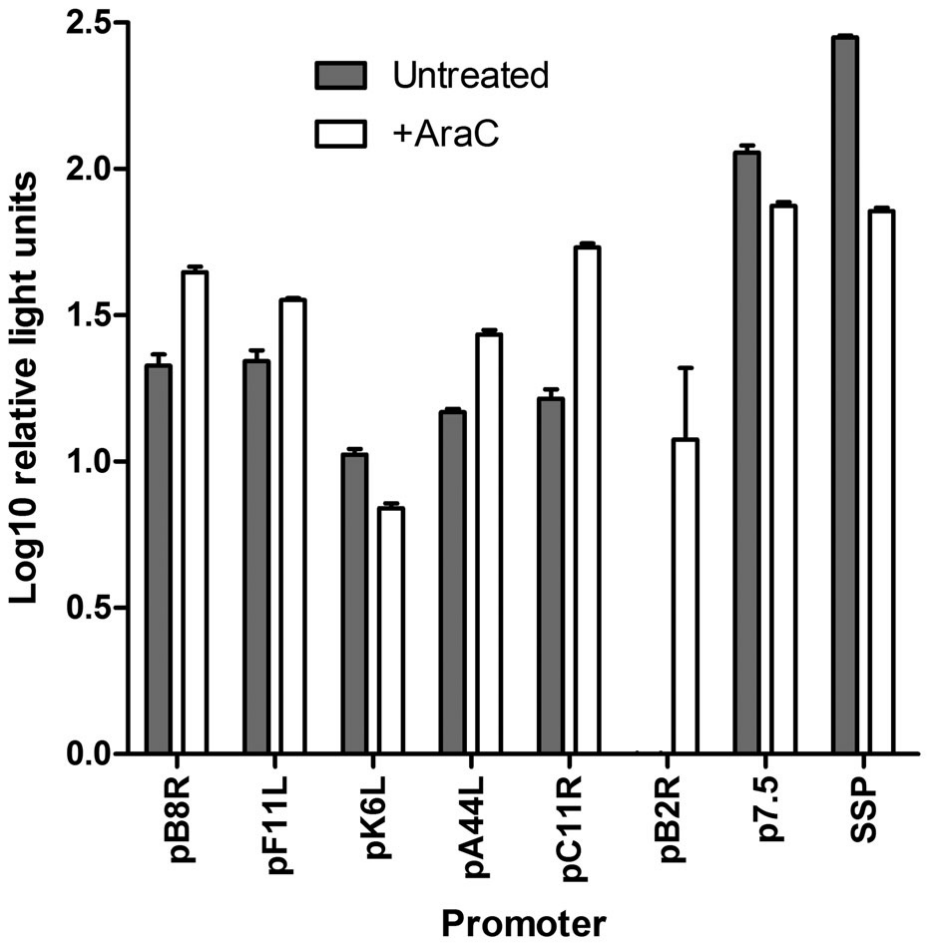
Activity of endogenous promoters compared to p7.5 and SSP *in vitro*. BHK cells were infected with 1 pfu/cell of recombinant MVA carrying tPA-Pb9-rLuc8PV under the control of the indicated promoters. Renilla luciferase activity in the culture supernatant was quantified at 8 h post-infection. Cells were either untreated (grey bars) or exposed to 40 µM AraC during and after infection (open bars), to inhibit post-replicative gene expression. Data shown are the mean and standard deviation of duplicates after subtraction of signal at 1 h post-infection and are representative of two independent experiments.

The tPA-Pb9-rLuc8PV transgene allows facile detection of renilla luciferase in the culture supernatant by virtue of the N-terminal tPA signal peptide and eight stability-enhancing point mutations [29]. All six of the selected promoters (Table 2) were able to drive expression of rLuc (Figure 2), which was measured 8 h after infection of BHK cells in the absence or presence of AraC to inhibit post-replicative gene expression. In the presence of AraC, the *B8R, F11L, A44L* and *C11R* promoters at their authentic loci (pB8R, pF11L, pA44L and pC11R) produced rLuc levels similar to that produced by p7.5 or SSP; however, in the absence of the inhibitor, the late promoter activity of p7.5 and SSP allowed higher protein expression. This was expected because p7.5 and SSP have combined early and late promoter activity, unlike the other promoters tested. In the case of pK6L and pB2R, the expression of rLuc was unexpectedly poor in both conditions: indeed, for pB2R, no luciferase was detected in the absence of AraC (the signal seen in the presence of the inhibitor at 8 hours post-infection in Figure 2 is likely due to prolonged, and therefore detectable, early gene expression in this condition).

### MVA Orthologs of K6L and B2R are Truncated and Fragmented Pseudogenes

Closer inspection of the sequences upstream of the MVA orthologs of *K6L (MVA026L)* and *B2R (MVA168R)* revealed fragmentation due to small deletions at the 5′ ends which we had overlooked during design of the constructs (Table 2). These mutations are found in addition to more obvious fragmentation in MVA, and the situation is complex because these genes are themselves fragmented in vaccinia virus compared to ancestral poxviruses such as cowpox virus (CPXV; NCBI RefSeq NC_003663.2). Vaccinia virus *K5L/K6L* (and *WR036*) are fragments of *CPXV045*; and vaccinia virus *B2R/B3R* are fragments of *CPXV197*. In MVA (GenBank U94848.1), these are annotated as the multi-ORF pseudogenes *MVA026L* and *MVA168R* and the smaller ORF remnants (*MVA027L* and *MVA169R*) do not feature in the current annotation at all, but are described only in the original paper [43]. We mistakenly targeted the initiation codons of *MVA027L* and *MVA168R* without realising until later that these do not contain the authentic *K6L* and *B2R* initiation codons, which are found further upstream (see Table 2). These ATGs lie much closer to the mean 40 bp distance from the early transcriptional start sites [19] and initiate severely truncated ORFs comprising the authentic N-terminal 14 plus 1 nonsense amino acids (*K6L*) or 30 plus 2 nonsense amino acids (*B2R*), as the result, in both cases, of a 20 bp deletion in MVA relative to vaccinia virus (positions 24691..24692 and 152186..152187 in U94848). Thus, it appears that these two genes, themselves fragments of larger cowpoxviral ORFs, are further inactivated in MVA by small frame-shifting deletions near their 5′ ends (and in the case of the *B2R* ortholog, even more fragmented into *MVA168/MVA169* by yet another deletion, of 14 bp). The use of the non-authentic ATG for insertion of tPA-Pb9-rLuc8PV (Table 2) presumably led to inefficient translation initiation and poor expression, suggesting that it is the upstream, non-annotated and severely truncated small ORFs containing the vaccinia virus start codons that are efficiently translated in MVA. The recombinant viruses employing the non-authentic initiation codons of *K6L* and *B2R* were not investigated further in this study.

### Kinetic Analysis of Transgene Expression *in vitro*

**Figure 3:**
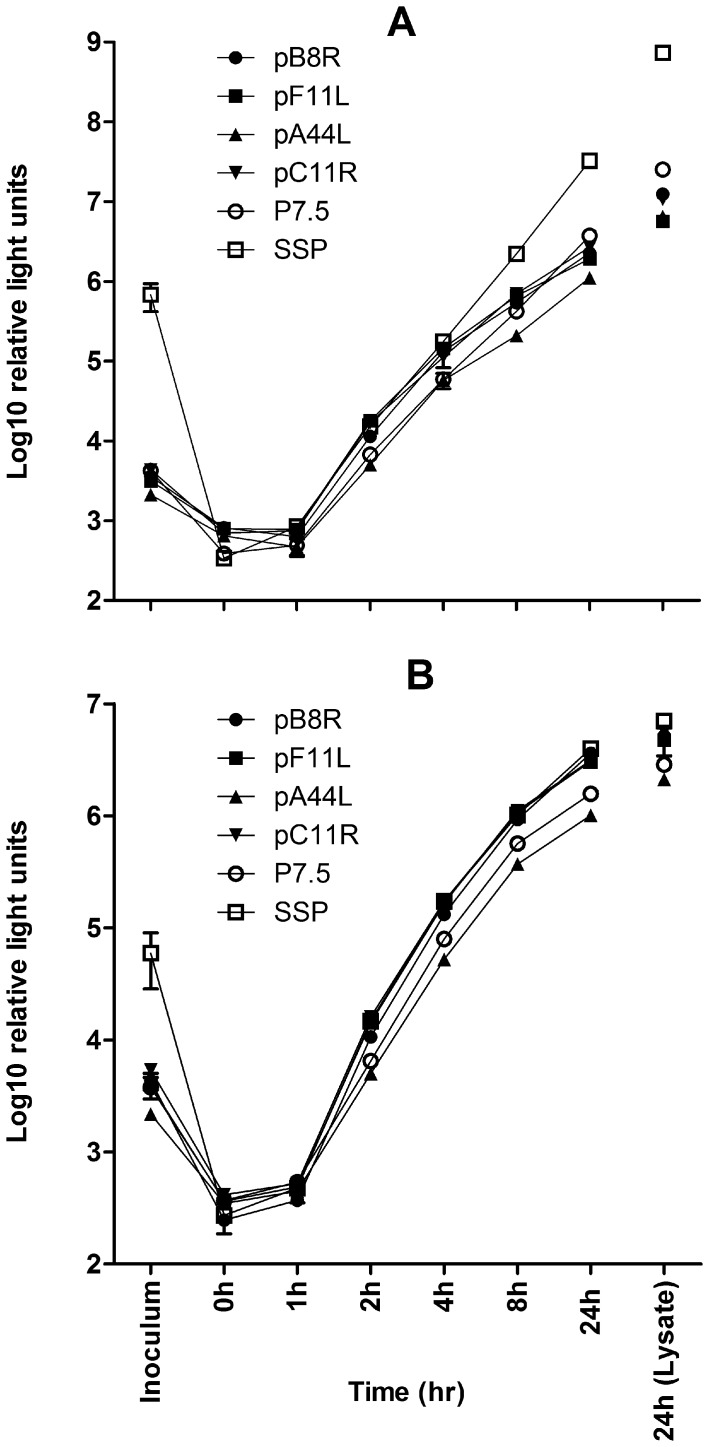
Timecourse of endogenous promoter activities compared to p7.5 and SSP *in vitro*. BHK cells were “spinoculated” (see Materials and Methods) with 1 pfu/cell of recombinant MVA carrying tPA-Pb9-rLuc8PV under the control of the indicated promoters. Renilla luciferase activity was quantified in the inoculum, the culture supernatant at various time points post-infection, and in the cell lysate at 24 h post-infection. Cells were either untreated (A) or exposed to 40 µM AraC during and after infection (B), to inhibit post-replicative gene expression. Data shown are the mean and standard deviation of duplicates and are representative of two independent experiments.

In measuring recombinant protein production by tPA-Pb9-rLuc8PV expressing viruses, we found that rLuc was readily detectable in the sucrose-concentrated viral preparations, especially in the case of SSP, the strongest late promoter. (The data in Figure 2 show the difference in rLuc levels from 1 h to 8 h post-infection; see legend). Although certain recombinant proteins [50] (and cellular proteins [51]) have been reported to be incorporated into various compartments of the vaccinia virion, we considered this was unlikely in the case of tPA-Pb9-rLuc8PV, since it is predicted to be a secreted, soluble protein. We therefore utilized a “spinoculation” [37] and washing protocol at 0°C before return to 37°C to allow viral entry. This effectively removed rLuc from the inoculum, confirming that its presence was indeed simply due to carry-over of soluble protein into the sucrose preparation, and allowing serial analysis of rLuc in the culture supernatant at various time points post-infection (Figure 3; compare “inoculum” to “0 h”).

Three of the four remaining endogenous promoters were remarkably similar to SSP in their ability to direct rLuc expression in the presence of AraC (Figure 3B), with p7.5 and pA44L exhibiting slightly weaker expression. When post-replicative expression was allowed, in the absence of the inhibitor, SSP drove very high levels of rLuc and the late promoter activity of p7.5 was also apparent (Figure 3A). Since pF11L, pA44R, pC11R and pB8R lack predicted late activity, the continued increase in rLuc levels at 8 – 12 h post-infection in the absence compared to the presence of AraC is likely the result of a second round of viral replication by progeny virus.

### Immunogenicity of Recombinant Antigen Driven by Endogenous Promoters

**Figure 4:**
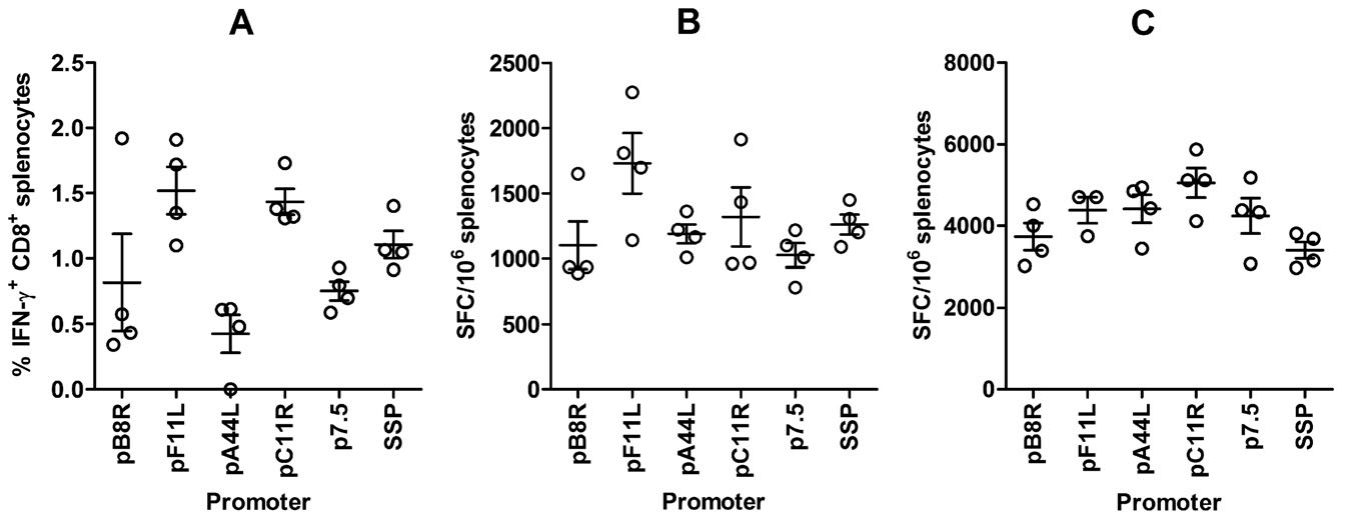
Cellular immunogenicity of recombinant MVA antigen (tPA-Pb9-rLuc8PV) driven by endogenous promoters compared to p7.5 and SSP in single-shot (A, B) or heterologous prime-boost (C) vaccination regimens. For single-shot (A, B), BALB/c mice were immunized i.m. with 10^6^ pfu rMVA) and splenic CD8^+^ T cell responses to Pb9 peptide were determined 7 days later by intracellular cytokine staining and flow cytometry (A) or IFN-γ ELIspot (B). For prime-boost (C), BALB/c mice were immunized i.m. with 10^8^ infectious units of AdCh63-tPA-Pb9-rLuc8PV and 56 days later received 10^6^ pfu rMVA. At day 70 (14 days post-boost), splenic CD8^+^ T cell responses to Pb9 peptide were determined by IFN-γ ELIspot. Circles represent the responses of individual mice, with lines indicating the mean and the error bars showing SEM. See text for statistical analysis. The data shown are representative of two independent experiments.

We used the Pb9 epitope fused to the N-terminus of our reporter construct tPA-Pb9-rLuc8PV to determine the ability of expression by pF11L, pA44R, pC11R and pB8R to elicit CD8^+^ T cells against the recombinant protein *in vivo*, in comparison to the traditional-style p7.5 and SSP promoter driven recombinants. One week after vaccination of mice with 10^6^ pfu rMVA, the frequencies of Pb9-specific CD8^+^ T cells were determined by intracellular cytokine staining (ICS) (Figure 4A) and IFN-γ ELIspot (Figure 4B). All the viruses elicited potent responses; and while by ELIspot there was no significant difference between the groups (p = 0.08 by one way ANoVA), pF11L and pC11R drove significantly higher CD8^+^ T cell frequencies than pA44L when the responses were measured by ICS (p<0.05 by Newman-Keuls post-test following one-way ANoVA; overall p = 0.005). In both readouts, and in other independent experiments (data not shown), the trend was the same, in that pF11L and pC11L elicited the highest CD8^+^ T cell frequencies, similar to SSP, with p7.5 and pB8R and A44L exhibiting slightly lower immunogenicity.

A heterologous prime-boost vaccination regimen employing a recombinant adenoviral vector followed 8 weeks later by rMVA expressing the same antigen is capable of eliciting extremely high frequencies of CD8^+^ T cells in mice [52], monkeys [34], [53] and humans [28]. We therefore performed a comparison of the endogenous promoters to p7.5 and SSP using this regimen, by priming with a chimpanzee adenovirus vector, ChAd63 [34], expressing the Pb9 epitope fused to GFP [35], and boosting at day 56 with rMVA. The frequencies of Pb9-specific CD8^+^ T cells were determined by IFN-γ ELIspot two weeks post-boost (Figure 4C). Surprisingly, SSP elicited the lowest frequency, and was statistically significantly worse than pC11R (p<0.05 by Newman-Keuls post-test following one-way ANoVA; overall p = 0.04), but the immune responses were not otherwise distinguishable. This may indicate that very abundant late gene expression may not be optimal for CD8^+^ T cell induction in an adenovirus-MVA prime-boost regimen; however, the data clearly show that all of the endogenous promoter driven insertion loci perform as well as the traditional p7.5 promoter in this context.

### Comparison of mH5 and p7.5 Promoters using tPA-Pb9-rLuc8PV Antigen

**Figure 5:**
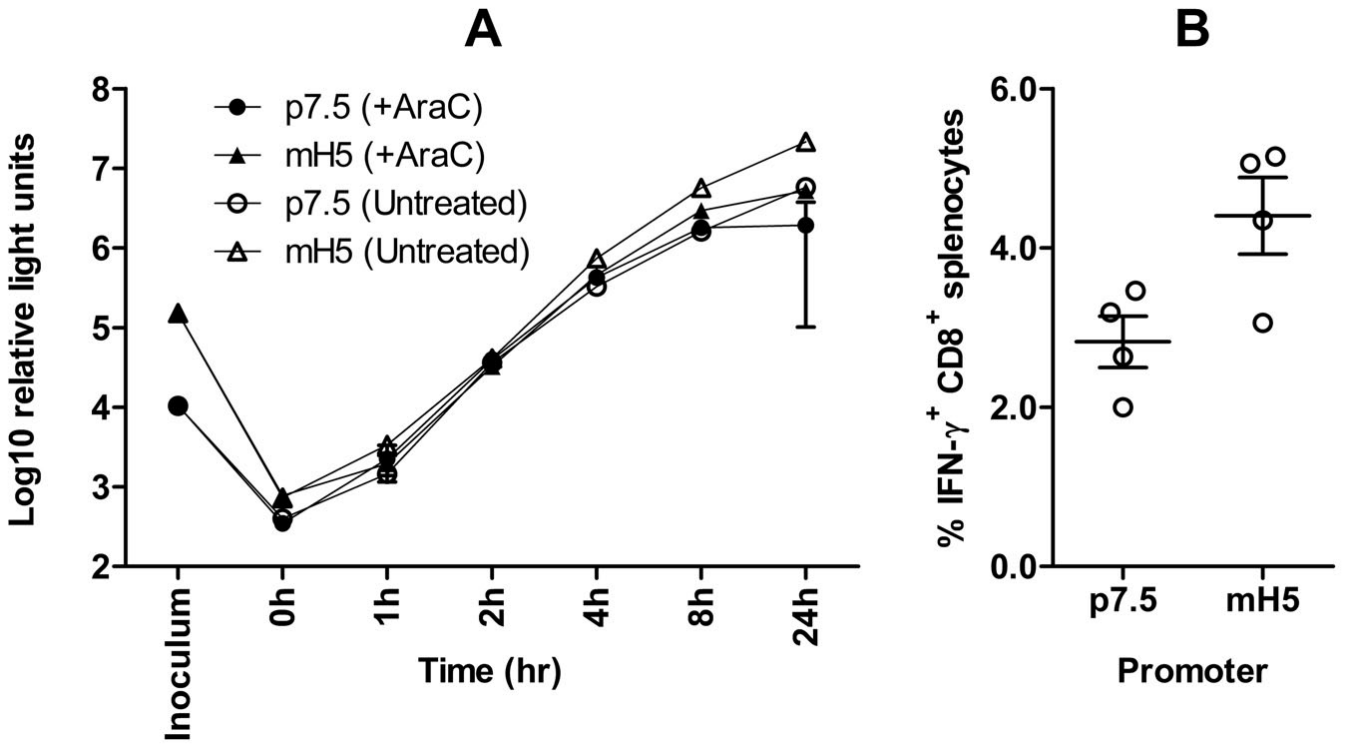
(A) Timecourse of activity of mH5 promoter compared to p7.5 *in vitro*. BHK cells were “spinoculated” (see Materials and Methods) with 1 pfu/cell of recombinant MVA carrying tPA-Pb9-rLuc8PV under the control of the indicated promoters. Renilla luciferase activity was quantified in the inoculum and then in the culture supernatant at various time points post-infection. Cells were either untreated or exposed to 40 µM AraC during and after infection as indicated, to inhibit post-replicative gene expression. Data shown are the mean and standard deviation of duplicates. The data shown are representative of two independent experiments. (B) Cellular immunogenicity of recombinant MVA antigen (tPA-Pb9-rLuc8PV) driven by mH5 compared to p7.5 in a single-shot vaccination regimens. BALB/c mice were immunized i.m. with 10^6^ pfu rMVA and splenic CD8^+^ T cell responses to Pb9 peptide were determined 7 days later by intracellular cytokine staining and flow cytometry. Circles represent the responses of individual mice, with lines indicating the mean and the error bars showing SEM. See text for statistical analysis. The data shown are representative of two independent experiments.

The mH5 promoter has enhanced early gene expression and cellular immunogenicity compared to p7.5 [11]. Although we did not perform a direct head-to-head comparison of the endogenous promoter driven insertion loci with a recombinant employing the mH5 promoter, we did evaluate the expression and immunogenicity of tPA-Pb9-rLuc8PV driven by mH5 compared with p7.5 when inserted as a traditional-style cassette at the TK locus by BAC recombineering. In agreement with what is to our knowledge the only other direct head-to-head comparison of murine CD8^+^ T cell induction by vaccination with recombinant MVAs employing the p7.5 and mH5 promoters [54], we observed a statistically significant increase in the frequency of Pb9-specific IFN-γ^+^ CD8^+^ T cells determined by ICS in the splenocytes of mice vaccinated with the mH5 construct (p = 0.03 by t-test) versus p7.5 (Figure 5B). Using our tPA-Pb9-rLuc8PV reporter system, we did not observe dramatic differences in levels of rLuc *in vitro* at early time-points post-infection, unlike the original finding with a β-galactosidase reporter [11] and another study using cytomegalovirus pp65 [55], though the augmented late promoter activity of mH5 was apparent (Figure 5A).

The frequencies of antigen-specific IFN-γ^+^ CD8^+^ T cells elicited by mH5-driven antigen were 1.56-fold higher than those elicited by p7.5-driven antigen (95% confidence interval [CI] from 1.06-fold to 2.07-fold) (Figure 5B). For the purposes of comparison with the flow cytometry data shown in Figure 4A, the ICS responses to pC11R-driven antigen were 1.90-fold higher (95% CI from 1.50- to 2.30-fold), and to pF11L-driven antigen 2.02-fold higher (95% CI from 1.43- to 2.61-fold), than those raised against p7.5-driven antigen.

### Transgene Insertion at Endogenous Promoter Driven Loci does not Adversely Affect Viral Growth

**Figure 6:**
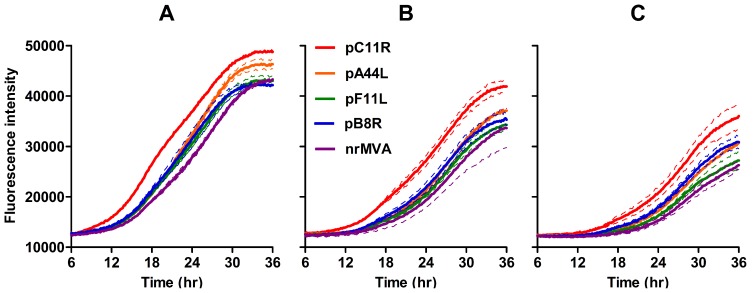
Growth rates of rMVA expressing tPA-Pb9-rLuc8PV under the control of endogenous MVA promoters in comparison to MVA lacking tPA-Pb9-rLuc8PV but containing the same GFP marker gene (nrMVA). BHK cells were infected with viruses at 1 pfu/cell (A), 0.5 pfu/cell (B) or 0.25 pfu/cell (C) and fluorescence of the viral GFP marker gene was quantified every 6 minutes for 36 h using a BMG FluoSTAR equipped with 37°C and 5% CO_2_ incubation. Thick lines show the mean of two replicates and adjacent thin lines of the same colour represent the standard deviation. The data shown are representative of two independent experiments. Fluorescence intensity is expressed in arbitrary units.

Although all the BAC-derived rMVAs expressing tPA-Pb9-rLuc8PV inserted to replace viral ORFs produced the expected viral yields during production (>10^9^ pfu/mL, see above), we nevertheless wished to measure the growth rate, since increasing inoculum or delaying harvest during propagation can compensate for reduced growth. All the BAC-derived rMVAs carried a separate GFP marker gene driven by the p4B late promoter of fowlpox virus [16], so we were able to use real-time fluorimetry as a proxy for viral growth rate. We have previously used this assay to show that derivation of MVA by the BAC method does not affect viral growth [32]. The growth curves of MVA lacking tPA-Pb9-rLuc8PV (but containing the GFP marker) and of the rMVAs employing pB8R, pF11L and pA44R to drive tPA-Pb9-rLuc8PV overlaid almost exactly (Figure 6), but rMVA using pC11R (i.e. lacking *C11R,* encoding the vaccinia growth factor) surprisingly exhibited a more rapid increase in GFP fluorescence and reached a slightly higher plateau. The viral yield after sucrose concentration did not differ significantly from that of other rMVA (3.7 × 10^9^ pfu/mL; see above). Nevertheless, we therefore conclude that insertion of the transgene at any of these four loci, concomitant with deletion of the targeted viral ORF, did not impair growth.

### Genetic Stability of rMVA with Transgenes Inserted at Novel Loci

**Figure 7:**
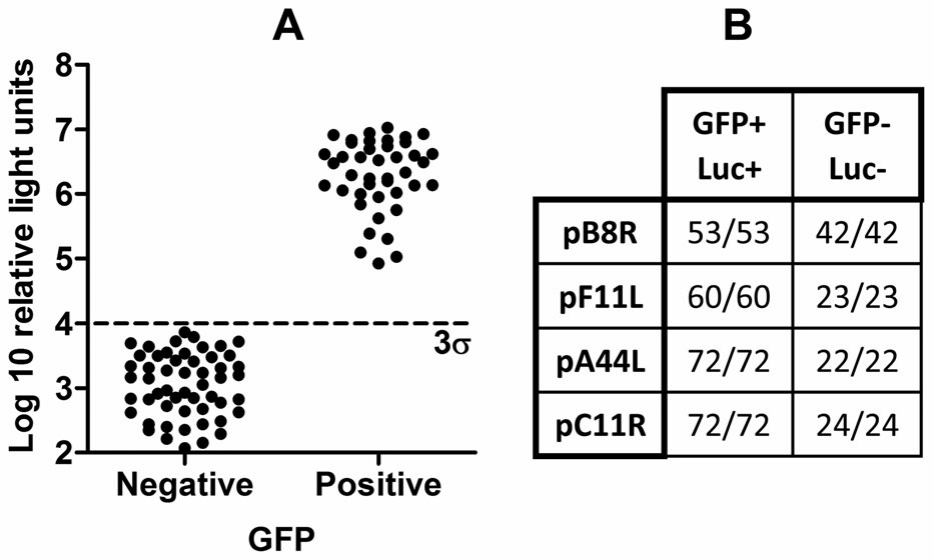
Genetic stability of rMVA expressing tPA-Pb9-rLuc8PV under control of endogenous promoters. Viruses were subjected to ten serial passages in CEFs, titred, and inoculated onto BHK cells at 0.001 pfu/cell. After 2 days, the cells were harvested and individually sorted into the wells of a 96-well plate using the CyCLONE attachment of a MoFlo flow cytometer. Two days later, renilla luciferase activity in the cell lysates was determined after scoring of wells as positive (+) or negative (−) for the viral GFP marker gene, indicating infection in the well. A cut-off of three standard deviations above the geometric mean of the GFP (dashed line labelled 3σ) was used to score GFP^+^ and GFP^−^ wells luciferase positive (Luc^+^) or negative (Luc^−^). Wells in which cell monolayers were lost during processing were excluded. Raw data for the pB8R recombinant (A) and well scores for all viruses (B) are shown.

Each of the four rMVAs employing the pB8R, pF11L, pA44L and pC11R promoters was passaged 10 times in CEFs at low multiplicity of infection. To verify that all viruses retained expression of tPA-Pb9-rLuc8PV, we flow sorted individual infected cells into microtitre plate wells, amplified the viruses by addition of BHK cells, and quantified rLuc expression in infected versus uninfected wells, which were differentiated via the viral GFP marker gene. Uninfected cells were the result of droplet loss during sorting or to lack of infectious progeny from GFP^+^ cells (the cells were lysed immediately after sorting). All GFP^+^ wells contained detectable rLuc (Figure 7), indicating that transgene insertion at these novel insertion loci does not in itself lead to genetic instability.

## Discussion

Here we show that using BAC recombineering it is straightforward to replace a non-essential poxviral ORF with a transgenic ORF and precisely retain the position of the initiation codon. This allows expression of the foreign protein by the promoter of the targeted gene, enabling assessment of the activity of the promoter at its natural locus, in terms of protein expression and immunogenicity. We demonstrate this in MVA using the orthologs of *C11R, F11L, B8R* and *A44L*, and compared early protein expression and immunogenicity with that achievable using the traditional p7.5 or SSP promoters coupled to the transgenic ORF and inserted at the thymidine kinase locus. Two promoters, pC11R and pF11L, enabled augmented CD8^+^ T cell responses compared to p7.5, and the magnitude of this difference was similar to the increment observed with mH5 compared to p7.5 in separate experiments. Furthermore, we demonstrate that insertion at all four of these sites does not result in genetic instability or impair viral growth or achievable titre.

Unexpectedly, the rMVA with the rLuc reporter gene inserted under control of pC11R, and therefore lacking the C11 vaccinia virus growth factor, exhibited a slightly faster increase in GFP fluorescence (expressed from a separate, late promoter driven transgene) during viral replication, despite having a very similar titre after amplification and sucrose concentration. C11 has been reported to activate NF-κB (S. Martin and J. Shisler, personal communication), so an absence of induction of cellular transcriptional programmes via this pathway might perhaps underlie this phenomenon. Since we have not performed traditional growth curves, we cannot conclude that this difference in GFP kinetics necessarily represents enhanced growth, but this does not affect the conclusion, reached in combination with the yield data, that the growth of MVA expressing a pC11L-driven transgene in place of *C11R* is unimpaired.

In two other cases *K6L* and *B2R*, we mis-positioned the insertion, and selected a downstream ATG (Table 2). The annotated initiation codons of *MVA026L* and *MVA168R* in the MVA genomic sequence (GenBank U94848.1) do not correspond to the authentic ATGs utilized in vaccinia virus *K6L* and *B2R*. Small MVA-specific deletions near the 5′ ends of these ORFs result in frame shifts, such that each authentic vaccinia virus ATG initiates a very severely truncated ORF of only 14 or 30 amino acids. Reporter gene expression using the 3′ (non-authentic) ATG was poor, indicating that these N-terminal polypeptide fragments are the only parts of the putative vaccinia virus B2 and K6 proteins that are efficiently expressed in MVA, presumably because translation initiation from the 5′ ATG is favoured, as previously reported [56]. These findings highlight the importance of positioning the ORF correctly relative to the promoter – one of the factors that likely impeded the uptake of endogenous promoter usage as a method for construction of recombinant poxviruses in the 1980s [1], [5]. Investigations of whether insertion at the authentic vaccinia virus ATG improves transgene expression at the *K6L* and *B2R* loci are currently on-going. We have not investigated whether intervening exogenous sequences lacking initiation codons, such as those derived from a multiple cloning site (as in our control p7.5 and SSP rMVAs and other traditionally-derived recombinants) affect transcription: this would not be expected based on current understanding of poxviral transcription [22]. It is intriguing to speculate on the mechanism of deletion at the *K6L* and *B2R* loci in MVA (there is a repeated 9 bp or 5 bp sequence separated by 20 bp at these loci in vaccinia virus, which may be relevant) and on the potential selective advantage of the severe truncation of K6 and B2 during derivation of MVA by serial passage – especially given that there is an additional fragmenting deletion in the *B2R* region of MVA, and that these vaccinia virus genes are themselves fragments of larger ancestral ORFs found (for example) in cowpox virus.

How well does the protein expression level we observed in MVA correlate with recent vaccinia virus transcriptomic data? We selected the promoters based on strong early mRNA expression in vaccinia virus microarray data [20], which has subsequently been complemented by a deep RNA sequencing study [19]. With regard to the four promoters investigated here, there is some disagreement both between and within these two studies, and with our protein-level data. The two methods were not unanimous in the clustering of *C11R, F11L, B8R* and *A44L* into the IE (E1.1) or E (E1.2) classes (Table 1), and we could not detect any meaningful difference in protein expression at the earliest time point (Figure 3). The rank order of mRNA abundance differed between early time points and an AraC treated group in the microarray, and within replicates in the deep sequencing study. Nevertheless, either *C11R* or *F11L* transcripts are the most abundant by both methods; and *A44L* transcripts consistently the least abundant, with those of other ORFs targeted here generally occupying an intermediate position – including p7.5, which was analysed at its natural locus (*WR001/C29L/B29R*). Thus, there is generally good agreement in the rank order of the promoter activities, as determined here by protein expression and immunogenicity (Figures 2, 3, and 4), and the abundance of mRNA from the driven vaccinia virus genes. However, there is some discrepancy in the magnitude of the difference. For example, there was an approximately 20-fold difference in mRNA copy number between the highest and lowest values for these five promoters; yet at the protein level (Figures 2 and 3) the range is much lower – threefold at most. This suggests that the promoter may not be the only factor affecting mRNA abundance in poxviruses: polyadenlyation or degradation rates could, for example, also play a role in a manner that is not apparent when an identical reporter gene is utilized.

CD8^+^ T cell responses to vaccinia virus are directed almost exclusively against early viral genes [39]–[42]. In agreement with this, we found that the cellular immunogenicity of rMVA employing early promoters was similar to those using early/late promoters (p7.5 and SSP), either as the sole immunogen or as a boost after priming with an adenoviral vector. The strongest promoters, pF11L and pC11R, elicited the highest frequencies of CD8^+^ T cell responses in the single-shot regimen. Two published approaches have enhanced the activity of early poxviral promoters: mutation of individual unfavourable nucleotides [23] of the core region of the *H5R* promoter, known as mH5 [11], and tandem insertion of multiple synthetic early promoter core regions, known as pHyb [57]. Consistent with our observations using wild-type pC11R and pF11L, both these interventions improve CD8^+^ T cell induction by a modest factor after a single rMVA vaccination compared to use of p7.5 [54], [57].

We did not perform a direct head-to-head comparison of mH5 with pC11R or pF11L, but we present our own comparison of p7.5 and mH5 in Figure 5. Use of mH5 to drive expression of recombinant antigen enabled about 1.5-fold higher CD8^+^ T cell responses compared with p7.5, versus about 2-fold using pC11R or pF11L compared with p7.5 in a separate experiment. Thus, it seems likely from this indirect comparison that pC11R or pF11L are at least as potent as mH5. Since the 95% confidence intervals of the improvements relative to p7.5 overlap (see Results), we conclude that further studies are required to establish the hierarchy more precisely.

We have also not yet investigated whether expression of a transgenic antigen from pC11R or pF11L modifies the pattern of immunodominance compared to viral antigens observed upon repeated immunization with rMVA, as reported for pHyb [57], nor whether the activities of these or similar endogenous promoters could be enhanced by rational modification, similar to mH5 [11], [23].An updated endogenous promoter driven transgenic strategy as described here has a number of potential advantages for generation of an rMVA-based vaccine product. If CD8^+^ T cells are the desired immune response, then late antigen expression is unnecessary, so promoters like those identified here, or variants thereof, would be ideal for maximal cellular immunogenicity but with minimal selective pressure resulting from excessive transgene expression due to strong late promoter activity *in vitro*, which can cause genetic instability [55], [58]. Alternatively, there are suitable early/late promoters in MVA that could be harnessed in the same manner, if late promoter activity were desirable, e.g. for induction of humoral responses against transgenic antigen. The use of endogenous promoters (with possible modifications) at their authentic loci may also offer an improved strategy for multivalent rMVA expressing multiple antigens from the same or different pathogens – another idea first demonstrated using vaccinia virus in the early 1980s [59]. Since homologous recombination is a major mutagenic mechanism in rMVA [14], introduction of the best conventional promoter, mH5, at multiple loci may inevitably carry an increased risk of instability, although such designs have been reported to be genetically stable [55].

To illustrate speculatively the potential applicability of the findings described here, two recent papers describing candidate ‘flu vaccines based on rMVA may be considered: one using haemagglutinin (HA) to induce protective antibodies in ferrets [60] and one using a nucleoprotein–matrix protein 1 (NP+M1) fusion protein to elicit T cell responses against these more conserved internal antigens in humans, potentially providing heterosubtypic immunity [61]. These studies used either the mH5 or p7.5 promoter inserted at the TK locus – the tried and tested, conventional approach. What would be the best design of an rMVA expressing two or more flu antigens, to combine these approaches? As an example, one could express HA traditionally, for example using mH5 at the TK locus, for antibody induction; and NP and M1 under control of pF11L and pC11L at their own loci, for CD8^+^ T cell induction. The identification of additional endogenous promoter driven insertion loci in combination with conventional approaches would allow expression of even more transgenic antigens. This could be valuable for development of new vaccines against more complex pathogens, for example, malaria parasites, where a multi-component vaccine targeting more than one stage of the life cycle is likely to be required to attain useful protective efficacy [62]. Even taking only the liver stage, it is already clear that vaccines that induce antibodies against the circumsporozoite protein or T cells against TRAP can each provide partial protection in humans, making them strong candidates for inclusion in a future combination vaccine [62].

Although viral vectored vaccines are showing promise in human clinical trials, it is also important that products have optimal immunogenicity and manufacturability. The application of BAC recombineering technology to poxviruses allows a precision of genetic manipulation that has the potential to allow realisation of ideas first conceived nearly 30 years ago.
